# Sexual health in the French military: a multidimensional and gendered perspective

**DOI:** 10.1186/s12889-018-5571-x

**Published:** 2018-06-18

**Authors:** Sandrine Duron, Aline Bohet, Henri Panjo, Nathalie Bajos, René Migliani, Catherine Marimoutou, Yann Le Strat, Jean Baptiste Meynard, Caroline Moreau

**Affiliations:** 1 French Military Center for Epidemiology and Public Health, Marseille, France; 2 INSERM, IRD, SESSTIM, Sciences Economiques & Sociales de la Santé & Traitement de l’Information Médicale, Aix Marseille Univ, Marseille, France; 3 Gender, Sexual and Reproductive Health, Centre for Research in Epidemiology and Population Health, (CESP), F-94807 Villejuif, France; 4 INED, F-75020 Paris, France; 5 French Military Medical Academy, Ecole du Val-de-Grâce, Paris, France; 6 Santé publique France, French national public health agency, F-94415 Saint-Maurice, France; 7 Department of Population, Family and Reproductive Health, Johns Hopkins Bloomberg School of Public Health, 615 North Wolfe Street, Baltimore, MD 21205 USA

**Keywords:** Sexual health, Prevention, Gender, Sexual risks

## Abstract

**Background:**

Sexual health in the military comprises a range of concerns including sexually transmitted infections (STI), unintended pregnancy, sexual violence and sexual dysfunction. This study aims to estimate the prevalence of sexual health concerns by gender in the French military and compare these prevalences to estimates in the general population.

**Methods:**

COSEMIL, the first sexual health survey in the French military comprises a probability sample of 1500 military personnel. Chi-square tests were used to compare lifetime abortion, STIs and sexual assault, and recent sexual dysfunction and sexual satisfaction by gender and explore the association between these indicators and current sexual risk (condom use at last intercourse).

**Results:**

Women were more likely than men to declare negative sexual health outcomes, with the greatest difference related to sexual assault (24.3% versus 5.1% of males, *p* < 0.001) and sexual dysfunction hindering sexuality (15.2% of females versus 5.3% of males, *p* < 0.001). Women were also twice as likely to report ever having an STI (6.7% versus 3.4%, *p* = 0.03). Comparison with the French general population indicates lower percentages of STIs among military men (2.9% versus 4.9%) and higher percentages of abortion (17.6% versus 14.3%) forced sex (10.6% versus 7.4%) and sexual dysfunction (14.2% versus 9.3%) among military women.

**Conclusion:**

These results highlight gendered pattern of sexual health in the French military with women suffering greater sexual risks than men. Military health services should include women’s health services to address the sexual and reproductive health gender gap.

## Background

According to the World Health Organization’s (WHO) working definition, sexual health is defined as “a state of physical, emotional, mental and social well-being in relation to sexuality” [1]. While the multidimensionality of sexual health is a recognized framework [2], its operationalization is still nascent, as most dimensions (infection, fertility, violence, dysfunction) are considered in silos and few studies include positive dimensions of sexual health. A growing body of research however, reports on the interrelation between sexual health dimensions, including violence, sexually transmitted infections (STI) and fertility control [2–7], but such an approach has yet to be developed in specific social contexts, such as the military, long recognized as a high risk population [8]. A focus on the “venereal peril” has guided much of the research and institutional response to sexual ill health in the military [9, 10]. The feminization of the military population along with a growing recognition of gender as a system of power regulating sexual interactions [11] has recently broadened research interest to other dimensions of sexual health in the military, including sexual coercion and fertility control [12, 13]. At the same time, the need to develop women’s health services is becoming increasingly evident in light of high levels of STIs and unintended pregnancies among active duty females in the US military [14–16].

Most of the evidence is generated in the US context and while knowledge regarding the different dimensions of sexual health expands, little is known about how these dimensions interconnect and how they relate to current sexual behavior [17]. The exploration of gender patterns in sexual health in the French military, one of the most feminized in Europe, is an opportunity to understand how gender inequalities vary by military context. As is the case in the US, the French military is a professionalized armed force, with increasing representation of women reaching 16% in 2016 [18]. Service men and women in France are younger than the general population, and have similar or higher levels of education and income, due to their employment status and qualification requirements (high school degree required at enrollment). Female recruits are less likely to hold a graduate degree and less represented at higher military ranks as compared to men [18, 19]. Within this population, we seek to 1) describe the prevalence of several dimensions of sexual health by sex and compare these prevalences to estimates in the general population 2) explore the intersection of these different dimensions and how they relate to current reports of condom non-use with a casual partner as an indicator of sexual risk.

## Methods

Data are drawn from the COSEMIL study (COMportement SExuel des MILitaires), conducted between 2014 and 2015. The goal of the COSEMIL study was to explore sexual norms and practices among a representative sample of the French military as well as different components of sexual health, including STIs, unintended pregnancies, sexual violence, sexual dysfunctions and sexual satisfaction.

COSEMIL followed a two-stage probability sampling design, based on the selection of 13 military units and 120 active duty members within each unit. Military units were randomly selected after stratification by location (Mainland France, or overseas bases) and military branch (Army, Navy, Air force), with unequal probabilities of selection based on unit size. Eleven units were selected in Mainland France (4 in the Army, 4 in the Navy and 3 in the Air Force) and one unit from each military branch was selected in Djibouti and French Guiana. Within each unit, 120 service members aged 18 and over were randomly selected with over representation of women (20% in our survey while women represented 16% of the armed forces in 2014). Based on pilot testing, we anticipated that 30 to 50% of individuals would not be available during the two-day data collection period carried out in each unit, due to scheduling conflicts. We therefore randomly drew additional replacements (40 to 60 replacements/ unit). Replacements were included only if the initially selected individuals were not available at the time of data collection. Upon selection, participants attended an information session describing study goals and procedures. They were informed about the voluntary nature of the study and provided written consent to participate. The COSEMIL survey received the approval of the French government oversight agency (Commission Nationale Informatique et Liberté, N° 2014–100).

Participants completed a 37-min average self-administered questionnaire on laptop computers and were ensured privacy by allowing sufficient distance between work stations to complete the survey. This questionnaire included information on socio-demographic characteristics and addressed a range of topics related to sexual norms, sexual lifestyles, sexual behaviors, contraceptive use and sexual health outcomes including history of STIs, abortion, sexual violence, sexual dysfunction and sexual satisfaction. The study examined other health related dimensions including substance abuse, mental health issues and general perceived health.

The present study focuses on the following five dimensions of sexual health.

- □ Lifetime history of STI was assessed with a question asking about ever having a sexually transmitted infection.
- □ Lifetime experience of abortion was assessed by asking female respondents if they had ever had an induced abortion (or for male respondents if they had a partner with whom they had an abortion).
- □ Lifetime Sexual assault, defined as an “unwanted physical contact involving sexual body parts” [20] was measured with a set of five questions, asking if the respondent ever had someone 1) “impose touching their sexual body parts” 2)“force them to touch someone else’s sexual body parts”, 3)“unsuccessfully attempt to force them to have sexual intercourse” 4)“force them to have sexual intercourse” or 5) “impose a sexual penetration of the vagina or the anus with a finger or object”. A four-item response option was used for all questions: “never”, “one time”, “several times”, or “it happened but don’t want to respond to further questions”. We defined three measures of lifetime experience of sexual violence:
- Forced sex: any experience of forced intercourse or sexual penetration with a finger or object.
- Attempted or Forced sex: any experience of forced sex or attempted forced intercourse.
- Sexual assault: any experience of forced sex, attempted forced sex or unwanted sexual contact.
- □ Sexual dysfunction causing a problem for the respondent’s sexuality in the last 12 months (henceforth abbreviated as *sexual dysfunction hindering sexuality)* was assessed through a set of five questions for females and six questions for males. These questions were the same as those used in the 2006 French national sexual health survey [21] and in the 2010 national sexual and reproductive health survey (FECOND study) [22]. The following symptoms were explored for both sexes: hypoactive sexual desire, orgasmic disorder, lack of pleasure and pain during intercourse. In addition, females were asked about vaginal dryness and males were asked about problems of erection and premature ejaculation. Response options were designed as a 4 point Likert scale ranging from often to never. Individuals, who reported a specific symptom (often or sometimes), were asked if the symptom “constituted a problem for their own sexuality”. Based on this information, we constructed a summary indicator of *sexual dysfunction hindering sexuality* coded 1 if respondents reported that they « often » experienced at least one symptom that caused a problem for their sexuality.
- □ Sexual satisfaction at the time of the survey was measured using a four-item response option: “very satisfied”, “somewhat satisfied”, “somewhat dissatisfied”, or “very dissatisfied”.

Current exposure to sexual risk was considered as a function of condom use according to type of partner (casual/regular) at last sexual intercourse. For women, condom use at last sexual intercourse was only considered in the context of last heterosexual sexual intercourse.

Unmet need for contraception was assessed by identifying women who were sexually active in the last 3 months, were not sterile or pregnant, did not wish to conceive and were not using any of the following methods of contraception at the time of the survey (pill, intra uterine device, implant, male or female sterilization, condom or other barrier methods, withdrawal or fertility awareness methods).

In this analysis, we first estimated the prevalence of sexual health indicators by sex in the military COSEMIL population aged 18 to 57 years. We then re-estimated these prevalences among a restricted sample of military recruits aged 18 to 49 years in order to compare sexual health indicators in the military to those derived from population based surveys conducted in France in 2010 (FECOND study) [23] and 2006 (CSF survey) [7]. The main features of these three surveys are presented in Table 1.

**Table 1:** Main methodological characteristics of 3 French surveys on sexuality

	CSF survey 2006	FECOND survey 2010	COSEMIL survey 2014–2015
Study population	Men and women from French General population	Men and women from French General population	Men and women from French armed forces
Age range	18–69 years	15–49 years	18–57 years
Sample size	12,364	8645	1500
Sampling method	Random digit dialing	Random digit dialing	2 stages random selection of participants based on human resources data
Data collection method	Telephone interviews (Questionnaire duration: 40 min on average)	Telephone interviews (Questionnaire duration: 41 min on average)	Self-administered surveys on laptop computers. (Questionnaire duration: 37 min on average)

We then evaluated bivariate associations between sexual health indicators and condom use at last sexual intercourse, as a marker of current sexual risk among men and women in the military. We used Chi2 tests corrected for complex samplign design. All analyses were weighted to account for complex sampling design and non-response. Analyses were performed using Stata 14 software.

## Results

**Fig. 1:**
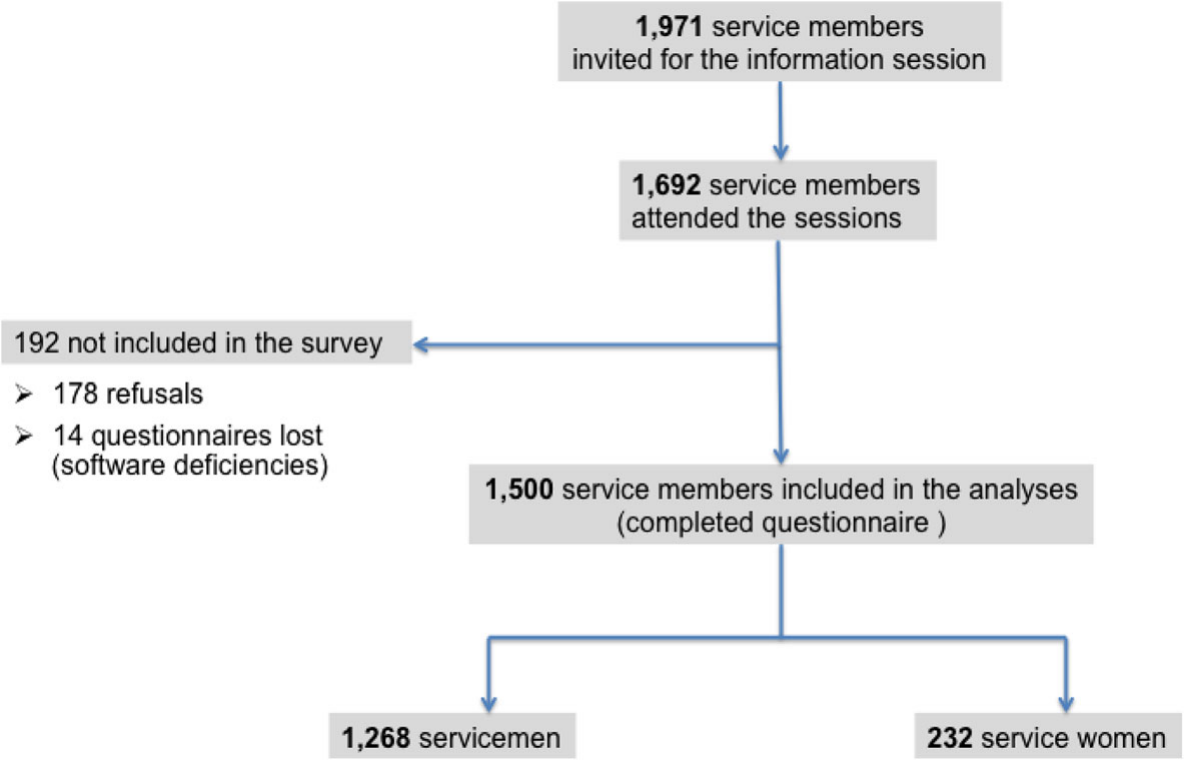
Flow chart of the COSEMIL study population

Altogether 1971 participants were invited to attend the information sessions, and 86% (*n* = 1692) attended. In all, 178 individuals refused to participate and 14 questionnaires were excluded due to software deficiency (Fig. 1). Our final sample comprised 1500 military recruits (76% of the 1971 were invited to participate) including 1268 males and 232 females. Comparing our study sample to the 115 individuals who refused to participate and completed a short refusal questionnaire, we found no significant difference in age, military rank, number of years in the military and deployment history but higher refusal rates in the Navy as compared to the Army or the Air Force.

The characteristics of the study population are described in Table 2. The mean age was 33.1 years for men and 30.6 years for women with a range from 18 to 57 years.

**Table 2:** Sociodemographic characteristics of French servicemen and women included in COSEMIL survey who have ever had sexual intercourse (*n* = 1482)

		Men		Women		
		n	%	n	%	*p*-value
Age	25 years	232	19.1	55	17.8	0.0004
	25–29 years	259	23.6	68	36.6	
	30–39 years	470	32.2	84	31.1	
	40 + years	289	25.1	25	14.5	
Current partner	Yes	1040	82.6	189	82.0	0.86
	No	210	17.4	43	18.0	
Marital Status	Married	536	38.9	61	25.3	0.002
	(PACS) Civil union	142	12.1	37	20.7	
	Single	511	44.8	120	48.1	
	Divorced	51	4.1	13	5.8	
	Widowed	2	0.1	0		
Level of education	No diploma or primary school	46	3.6	5	3.2	0.03
	<High school	483	38.7	64	26.8	
	High school graduation	464	34.7	114	46.1	
	Bachelor’s	190	16.7	38	19.0	
	Graduate degree	65	6.4	10	4.9	
Place of birth	Mainland France	1091	86.8	202	88.6	0.67
	Overseas France	114	9.7	23	8.0	
	Foreign country	45	3.6	7	3.4	
Number of children	0	545	44.8	140	62.5	0.001
	1	232	17.7	38	13.3	
	2	318	23.9	45	20.0	
	> = 3	153	13.6	9	4.2	
Military branch	Army	572	61.6	73	39.6	< 0.001
	Air force	375	18.4	97	43.6	
	Navy	303	20.0	62	16.8	
Military rank	Officer	117	12.2	14	6.3	0.002
	Noncommissioned Officer	584	45.1	99	46.8	
	Enlisted personnel	549	42.8	119	46.9	
Length of service	<=5 years	298	26.1	67	28.5	0.0003
	5–15 years	461	34.9	115	46.5	
	15–25 years	356	27.0	43	22.8	
	> 25 years	131	12.1	7	2.2	
Military status	Military carrier	474	37.7	48	21.9	< 0.001
	Under contract	775	62.3	184	78.1	

A total of 1250 males and 232 females reported ever having sexual intercourse. Table 3 presents the distribution of sexual health indicators by sex among the service members included in COSEMIL survey. A vast majority indicated that they were satisfied or very satisfied with their sexual lives, with no difference by sex. However, women were systematically more likely than men to declare a range of negative sexual health outcomes, with the greatest sex-difference related to sexual violence (24.3% of females reported ever experiencing sexual assault versus 5.1% of males, *p* < 0.001) and sexual dysfunction hindering sexuality (15.2% of females versus 5.3% of males, *p* < 0.001). Women were also twice as likely to report ever having an STI than men (6.7% versus 3.4%, *p* = 0.03).

**Table 3:** Frequency of Sexual health outcomes by sex, among participants who ever had sexual intercourse, according to the survey (COSEMIL, FECOND and CSF surveys)

	COSEMIL survey			COSEMIL survey			FECOND survey			CSF survey		
				Standardized by age on general population (18–49 years)			General population restricted to age range18–49 years			General population restricted to age range 18–49 years		
	Men	Women	*p*-value	Men	Women	*p*-value	Men	Women	*p*-value	Men	Women	*p*-value
	*n* = 1250	*n* = 232		*n* = 1217	*n* = 228		*n* = 2681	*n* = 4283		*n* = 4397	*n* = 5202	
Lifetime Sexually transmitted infections (STI) reported	3.4%	6.7%	0.03	2.9%	6.4%	0.02	4.9%	8.1%	0			
	[2.3–4.9]	[4.5–9.8]		[1.9–4.4]	[4.1–10.0]		[4.1–5.9]	[7.3–9.0]				
Lifetime abortion	13.6%	20.0%	0.002	14.2%	17.6%	0.24	11.1%	14.3%	0.003			
	[10.1–17.9]	[16.9–23.4]		[9.9–19.9]	[15.2–20.4]		[9.9–12.6]	[13.1–15.5]				
Lifetime experience of sexual assault^a^												
* Forced sex*	2%	11%	< 0.001	3.1%	10.6%	< 0.001				1.5%	7.4%	< 0.001
	[1.6–2.6]	[7.3–16.3]		[2.0–4.7]	[7.5–14.6]					[1.1–2.0]	[6.5–8.3]	
* Forced/attempted sex*	3.4%	12.7%	< 0.001	4.4%	12.1%	< 0.001				4.8%	17.3%	< 0.001
	[2.5–4.5]	[8.2–19.1]		[3.3–5.9]	[9.2–15.9]					[4.1–5.7]	[16.1–18.5]	
* Sexual assault*	5.1%	24.3%	< 0.001	6.2%	22.6%	0.005				6.6%	21.7%	< 0.001
	[3.7–7.0]	[17.4–32.9]		[4.5–8.5]	[12.0–38.3]					[5.7–7.6]	[20.4–23.0]	
Sexual Dysfunction in the last 12 months	5.8%	15.5%	0.008	6.3%	14.5%	0.003	6.7%	12.1%	< 0.001			
	[4.4–7.8]	[9.3–24.9]		[4.5–8.9]	[8.3–23.2]		[5.6–7.9]	[11.2–13.3]				
Sexual Dysfunction causing distress the last 12 months	5.3%	15.2%	0.006	5.6%	14.2%	0.003	3.6%	9.3%	< 0.001			
	[3.8–7.3]	[9.0–24.6]		[3.9–8.0]	[8.3–23.2]		[2.8–4.6]	[8.4–10.2]				
Sexual Satisfaction	85.7%	86.9%	0.57	86.3%	87.9%	0.65	89.4%	88.7%	0.42			
	[82.7–88.2]	[82.5–90.4]		[83.6–88.6]	[79.8–93.0]		[88.1–90.5]	[87.6–89.7]				

^a^Lifetime experience of sexual assault is calculated among all males (*n* = 1268), since 2 of the 18 males who never had sex, reported having been the victims of sexual assault

Exploring the interconnection between the different negative dimensions of sexual health among the military (STIs, forced sex, abortion and sexual dysfunction causing distress), we found that women were more likely than men to report any unfavorable sexual health experience (39.4% versus 21.7%, *p* < 0.001) and more likely to report at least two unfavorable outcomes as compared to men (11.7% versus 2.0%, *p* < 0.001).

Comparison of the military COSEMIL population (aged 18–49 years) with the French general population of adults of the same age indicates lower percentages of reported STIs among men in the military (2.9% [1–9-4.4] versus 4.9% [4.1–5.9]) and higher percentages of military women reporting an abortion (17.6% [15.2–20.4] versus 14.3% [13.1–15.5]). Women in the military were also twice as likely than the French female population to report often experiencing sexual dysfunction hindering sexuality (Table 3). We found a slightly higher percentage of men and women in the military who reported lifetime experience of forced sex as compared to the general population (3.1% versus 1.5% for men and 10.6% versus 7.4% for women) but no difference in lifetime experience of sexual assault. Service members were equally likely to indicate a satisfying sexual life as the general population.

Among military respondents who had sexual intercourse in the last 12 months (Table 4), the same proportion of men and women indicated that their last partner was a casual partner (12.8% of men and 13.3% of women, *p* = 0.87). Service men and women were equally as likely to report using a condom at last sexual intercourse (18.9% of women and 20.3% of men, *p* = 0.66). However, service-women were more likely than service-men to report non-use of a condom at last sex with a casual partner (7.3% versus 4.1%, *p* = 0.006). Only a minority of service members reported not using contraception while at potential risk of unintended pregnancy (1.5% of men and 2.4% of women, *p* = 0.30).

**Table 4:** Condom use at last sexual intercourse by sex, according to the type of partner and among participants who reported intercourse in the last 12 months, COSEMIL survey and FECOND survey

	COSEMIL 18–57		COSEMIL 18–49		FECOND 18–49	
	Men	Women^a^	Men	Women^a^	Men	Women^a^
	*n* = 1199	*n* = 191	*n* = 1153	*n* = 187	*n* = 2591	*n* = 4097
Casual partner at last sex						
	12.8%	13.3%	11.5%	10.6%	9.3%	4.4%
	[10.6–15.3]	[9.4–18.5]	[9.4–14.4]	[7.9–14.0]	[8.3–10.5]	[3.8–5.1]
Condom use at last sex as a function of type of partner						
Condom used	18.9%	20.3%	15.5%	19.3%	29.1%	18.5%
	[17.6–20.2]	[14.1–28.4]	[13.7–17.4]	[12.5–28.7]	[27.2–31.0]	[17.2–19.8]
No condom used with regular partner	77.1%	72.4%	80.2%	74.4%	69.5%	80.3%
	[75.5–78.6]	[65.2–78.6]	[77.5–82.6]	[66.8–80.8]	[67.5–71.3]	[79.0–81.6]
No condom used with casual partner	4.1%	7.3%	4.4%	6.3%	1.5%	1.2%
	[3.2–5.17]	[5.0–10.4]	[2.9–6.5]	[4.0–9.6]	[1.1–2.0]	[0.9–1.6]
Unmet need for contraception	1.5%	2.4%	1.7%	4.8%	2.2%	2.4%
	[0.7–2.9]	[1.2–4.6]	[0.9–3.4]	[1.3–15.9]	[1.7–2.9]	[2.0–3.0]

^a^ women who had sexual intercourse with another woman at last sexual intercourse were not included in this analysis

Compared to the general population, military personal aged 18–49 years old were more likely to report their last partner was a casual partner (Table 4). Both men and women in the military were more likely to report unprotected intercourse with a casual partner at last sexual intercourse (Table 4). While the frequency of unmet need among women 18 to 49 years in COSEMIL was higher than in the general population (4.8% versus 2.4%), our confidence interval is too large to draw any inference (Table 4).

Associations between different domains of sexual health among the military population are depicted in Table 5. Results indicate that military personnel who were unsatisfied with their current sexual life and those who reported sexual dysfunction hindering sexuality in the last 12 month were less likely to use a condom at last sex with a casual partner (Table 5).

**Table 5:** Relationship between sexual health indicators and current sexual behavior in the form of condom use at last sexual intercourse, among service members who reported intercourse in the last 12 months, COSEMIL survey

Use of condom at last sexual intercourse						
		1) Condom used	2) No condom used regular partner	3) No condom used casual partner	*p*-value	*p*-value
						(3 vs (1 + 2))
Men						
Total		18.9%	77.1%	4.1%		
Sexual dysfunction causing distress	yes	17.3%	74.0%	8.7%	0.14	0.08
	no	18.9%	77.3%	3.8%		
Sexual Satisfaction	yes	16.6%	80.5%	2.9%	0.004	0.004
	no	33.7%	53.9%	12.3%		
Women						
Total		20.3%	72.4%	7.3%		
Sexual dysfunction causing distress	yes	2.6%	78.3%	19.1%	0.003	0.006
	no	23.5%	71.4%	5.1%		
Sexual Satisfaction	yes	18.3%	77.6%	4.2%	0.003	0.002
	no	33.6%	39.0%	27.4%		

## Discussion

Results of this first national sexual health study in the French military exhibits a gendered pattern of sexual health with women suffering greater sexual risks than men with regards to STI, sexual violence and sexual dysfunction. Women were nearly twice as likely to report any sexual health concern than men and were more likely to report several concerns (30% of women who indicated any concern reported at least two versus 10% of men). These findings corroborate the findings of the 2008 Department of Defense Survey in the United States indicating greater self-reports of STIs, unwanted sexual contact among servicewomen as compared to servicemen [24]. Gender differences in *Chlamydia Trachomatis* rates are also noted in a study using US army veterans’ medical records [25] or in a study using systematic screening for US army soldiers deployed in Korea [26]. Studies on military sexual trauma in the US also consistently report higher incidence and prevalence rates of sexual assaults among women as compared to men [15, 20]. Few studies explore gender differences in sexual functioning in the military context, with most of the research focusing on the psychological underpinnings of sexual dysfunction as it relates to posttraumatic stress disorder. Nevertheless, our results show higher prevalence of sexual dysfunction among women than men, mirroring findings from the 2006 French sexual health survey [21]. The substantial gender gap across a range of sexual health concerns [14, 27, 28] has led to the development of women health service in the US military, among veterans [29] as well as in the US Navy, through the Sexual health and responsibility program (SHARP) [16]. French Armed forces have yet to develop such a programmatic approach. Further analysis of the intersection of gender with other salient dimensions of social hierarchy, including social class and military rank are underway to shed light on the social determinants of sexual health and its linkages to other aspects of physical and mental health in order to inform the integration of sexual health in primary healthcare services in the French military.

After standardizing for age, our study reveals little difference in self-reports of STIs in the military as compared to the general population in France, a slightly higher frequency of forced sex as well as increased prevalence of abortion among women. We also found a lower frequency of condom use with a casual partner among military personnel. In addition, sexual dysfunction hindering sexuality was more prevalent in the military as compared to the general population. Several interrelated mechanisms are evoked to explain differentials in sexual risks among army recruits as compared to civilians: population composition, sexual behavior and sexual networks [30, 31]. Army recruits are traditionally younger, less likely to be married and in some contexts, more socio-economically disadvantaged. In our study, we adjusted for age and found little differences in educational level (as a marker of socio-economic status) between the military and the French general population, which may contribute to our findings of similar prevalence of STIs. However, we also found higher frequency of forced sex compared to CSF survey estimates in 2006, which calls for a more thorough investigation of sexual violence in the military context, where hegemonic masculinity ideology prevails [11, 32, 33]. Our results are also higher than the latest French national estimates based on VIRAGE population based survey conducted in 2015, indicating 0.61 and 3.72% lifetime experience of forced sex for males and females aged 20–69 years [34]. These later comparisons should be considered with caution given differences in age range and survey instruments. A number of studies report low use of condoms in military populations [35], which we further specify by showing that military personnel report lower use of condoms with casual partners. Lack of condom use with a casual partner was not related to a history of STI or abortion in our study, but we found a strong association with sexual satisfaction and sexual dysfunction among women. The connection between pleasure-related attitudes and condom use has been described in the general population in the US [36], while the association between sexual dysfunction and history of STI was reported in the national sexual Health Survey in Britain [5]. Given the higher prevalence of sexual dysfunction in our military population, our results call for a more holistic approach to sexual education and counseling that addresses issues of sexual dysfunction and promote positive attributes of sexuality as a way to improve safe sexual practices [37].

Safe sexual practices are dependent on interactions between partners, calling for greater attention to the relational and situational context in which sexual interactions take place. A recent study on STIs and sexual behavior of shipboard US military personnel indicates that most STI transmission occurs within a constricted sexual network involving military spouses and non-spousal relationships between military personnel. The study reports frequent unprotected sexual interactions between service members in non-spousal relationships, which may explain our findings of less frequent use of condoms with casual partners in the military as compared to the general population. In addition, a number of studies highlight the high prevalence of substance abuse in the military and its connection with a range of sexual risks [38–40]. Further analysis of the relational aspects of sexual interactions in the military as well as the connection between several risky behaviors, including alcohol abuse, is underway, in order to inform the integration of sexual health within primary care for men and women in the military.

Our study has a number of limitations. First, we rely on self-reports of sexual health outcomes, such as STIs, abortion or sexual violence that are underreported in population based studies, due to social stigma or lack of recognition of asymptomatic infections. An ongoing analysis regarding the differential estimates of reported and prevalent diagnostic cases of STIs (based on biomarkers) will shed light on the factors related to STI reporting versus STI diagnosis in this population. While underreporting of sexual outcomes is a limitation, the same issues arise in the FECOND survey, from which we draw our comparisons [22]. In addition, the use of the same study instruments in the COSEMIL and population based surveys (2006 CSF [7, 21] and 2010 FECOND survey [22]) limits differential measurement error. The impetus of comparing the COSEMIL population with the French general population as well as the length of the COSEMIL questionnaire informed our choice of survey indicators. As a result, we did not use validated measures of sexual functioning, such as the Female Sexual Functioning Index [41] or the Brief Sexual Function Inventory [42], which include many more questions than our instrument. However, our measure captures all the dimensions of sexual dysfunctions assessed in these scales and is more in line with the most recent definition of sexual dysfunction from the 5th edition of the Diagnostic and statistical manual of mental disorders (DSM-V), involving symptomatology causing significant distress for a prolonged period of time [43]. Finally, the cross-sectional nature of COSEMIL survey prevents any causal inference in the associations described, particularly with respect to the relations between the different domains of sexual health.

## Conclusion

In conclusion, this first national sexual health survey conducted in the French military highlights the gendered pattern of sexual health in the French military. Service women are at heightened risk of sexual health problems as compared to service men and to women in the general population. These results should incentivize the French military to integrate women’s health services to primary care services in the military and expand counseling on a wider range of Sexual Reproductive Health topics, such as violence or dysfunctions that are prevalent and inform sexual risk raking.

## Abbreviations

COSEMIL: COMportement SExuel des MILitaires
DSM-V: 5th edition of the Diagnostic and statistical manual of mental disorders
SHARP: Sexual health and responsibility program
STI: Sexually transmitted infection
